# The role of shame in the relationship between bullying and self-harm in portuguese adolescents

**DOI:** 10.1192/j.eurpsy.2021.589

**Published:** 2021-08-13

**Authors:** C. Bento, A.T. Pereira, M. Oliveira, T. Cartaxo, F. Carvalho, A. Macedo

**Affiliations:** 1 University Clinic Of Paediatrics, University of Coimbra/Faculty of Medicine, Coimbra, Portugal; 2 Institute Of Psychological Medicine, Faculty Of Medicine, University of Coimbra, coimbra, Portugal; 3 Pediatric Hospital Of Coimbra, Coimbra Hospital and Universitary Centre, Coimbra, Portugal; 4 Institute Of Psychological Medicine, Faculty Of Medicine, University of Coimbra, Coimbra, Portugal

**Keywords:** Portuguese adolescent girls, Bullying Victimisation, Suicidal ideation

## Abstract

**Introduction:**

Bullying consists in acts of intentional and repeated physical or psychological violence, completed by an individual or a group of individuals, provoking pain, distress and shame. Currently, it is considered a serious problem with health implications in adolescents. Shame is a self-conscious, multifaceted and socially focused emotion that relates to a negative self-assessment.

**Objectives:**

The study aim was to investigate the mediating role of Shame in the relationship between Bullying and Self-harm and Suicide Ideation in Portuguese adolescents.

**Methods:**

346 adolescents (58.4% girls), aged 15.32±1.193 from public and private schools (9^th^ to 12^th^ grades) in Coimbra, answered the validated Portuguese versions of the Bullying Questionnaire, the Other as Shame Scale for Adolescents and the Self-Harm and Suicidal Ideation Questionnaire. For data analysis the SPSS 26 and Macro Process (Hayes 2020) was used.

**Results:**

Bullying Victimization had a prevalence of 18.78%. Girls and boys significantly differ in Bullying Victimisation, Self-Harm, Suicidal Ideation and Shame mean scores (all p<.05). In girls, Bullying Victimisation was correlated with Shame and Suicidal Ideation. The mediation analysis showed that, in girls, Shame partially mediated the relationship between Bullying and Suicidal Ideation (p<.001). We didn’t find these results in boys.

**Conclusions:**

Bullying is a global problem that needs to be addressed. Adolescents of today are the adults of tomorrow. In a physical growth and mental maturation phase, it is urgent to avoid disruptors which lead to psychopathology. Our results corroborate that Shame can be a harmful factor in Bullying with deleterious consequences in adolescents.

